# Effects of exercise training on oxidative phosphorylation-related genes in a diabetic heart via microarray analysis

**DOI:** 10.1186/s12872-025-05496-9

**Published:** 2026-02-02

**Authors:** Iqbal Ali Shah, Shahid Ishaq, Shin-Da Lee, Bor-Tsang Wu

**Affiliations:** 1 PhD Program in Healthcare Science, College of Healthcare Science, China Medical University, Taichung, Taiwan; 2 Department of Physical Therapy, China Medical University, Taichung, Taiwan; 3 Department of Senior Citizen Service Management, National Taichung University of Science and Technology, Taichung, Taiwan

**Keywords:** Diabetic heart, Mitochondrial complexes (I, III, IV, V), Gene expression, Molecular pathways, Oxidative phosphorylation, Physical activity

## Abstract

**Background:**

Diabetic heart disease is marked by structural, functional, and molecular alterations in the myocardium. We investigated the effects of exercise training on oxidative phosphorylation-related genes in the diabetic heart.

**Methods:**

Male mice C57BL/6JNarl (*n* = 24) were randomly divided into three equal groups: STZ-induced diabetic group with 12-week exercise training (DM-EX); diabetic group (DM); a non-diabetic control group. After completion of exercise training, samples from the soleus and heart tissues were collected from all the mice. Out of eight mouse samples from the DM and DM-EX, two left ventricles from either group were randomly selected and processed for RNA extraction and microarray analysis.

**Results:**

Exercise training changed the expression of 517 genes (*P* < 0.05); 380 upregulated,137 downregulated. Enrichment analysis depicted that apoptosis, diabetic cardiomyopathy, and oxidative phosphorylation were most significantly regulated. Pathway analysis revealed that exercise training upregulated 17 key oxidative phosphorylation genes, including complex I (Ndufa13, Ndufb4, Ndufb8, Ndufb9, Ndufc1, Ndufc2, Ndufs4, Ndufv2), complex III (Uqcrfs1, Uqcrh), complex IV (Cox5A, Cox7A, Cox17), and complex V (Atp5e, Atp5g3, Atp5k, Atp5l).

**Conclusion:**

Exercise training amplifies the gene expression concerned with oxidative phosphorylation in the diabetic heart, showing its potential to modulate molecular pathways that influence cardiac functions and improve the diabetic heart.

**Clinical trial number:**

Not applicable.

## Introduction

The occurrence of cardiac dysfunction has been reported to be around 35.5% in Type-2 and 14.5% in Type-1 diabetes, respectively [1]. Poor glycemic control makes a significant contribution to the risk and development of diabetic heart disease (DHD) and subsequent heart failure [2]. DHD encompasses various conditions, including diabetes autonomic neuropathy, diabetic cardiomyopathy, and coronary arterial dysfunction, each with distinct functional, structural, and molecular remodeling of the myocardium [3]. Of these, diabetic cardiomyopathy uniquely manifests as myocardial dysfunction independent of atherosclerosis, coronary artery disease, and hypertension, as shown by preclinical and clinical trials [4]. Notably, heart failure risk is increased by 2.5 times in men and five times in women with diabetes, irrespective of age or other cardiovascular comorbidities [5]. DHD pathogenesis is multifactorial, driven by altered substrate utilization, impaired calcium homeostasis, chronic inflammation, defective insulin signaling, neurohormonal activation, mitochondrial dysfunction, oxidative stress, dysregulated gene expression, and cardiomyocyte death [6, 7]. Constant inflammation and elevated oxidative stress promote diabetic heart disease by enhancing the generation of reactive oxygen species (ROS) and accelerating atherosclerotic changes at the vascular level [8].

Cardiac tissue needs a continuous and substantial supply of energy to maintain contraction, relaxation, and ionic balance [9]. Around 95% of adenosine triphosphate (ATP) generated in cardiac cells is through mitochondrial oxidative phosphorylation [10]. The derivatives of the citric acid cycle, nicotinamide adenine dinucleotide (NADH) and flavin adenine dinucleotide (FADH2), donate electrons to complex I and II, respectively. Passage of electrons across complexes I-IV drives proton shuttling across the inner mitochondrial membrane, and the resulting electrochemical gradient powers complex V (ATP synthase) to convert ADP to ATP, with water formed as a byproduct [11]. A diabetic heart has decreased myocardial glucose utilization because of insulin resistance, decreased level of glucose transporters such as GLUT4, and reduced pyruvate dehydrogenase activity. Therefore, diabetic heart mainly depends on the oxidation of fatty acids for ATP production, which is associated with reduced mitochondrial respiratory efficiency [12, 13]. Previous studies have reported impaired state 3 respiration, lower ATP generation, and evidence of insulin resistance in cardiac mitochondrial tissue from prediabetic and diabetic models [14, 15]. In addition, the genes encoding mitochondrial oxidative phosphorylation are downregulated in the diabetic heart [12].

Exercise training markedly enhances myocardial mitochondrial dynamics and biogenesis, optimizing substrate utilization, lowering myocardial fibrosis, and promoting autophagic flux, collectively contributing to improved myocardial energy metabolism and decreased mortality associated with diabetic heart disease [16]. Resistance and aerobic exercise enhance insulin sensitivity and endothelium-mediated vasodilation, increase cardiac tissue velocity, oxygen uptake, and ejection fraction, ultimately improving myocardial activity in diabetic heart disease [17]. Early exercise intervention in diabetic mice stimulates the production of vascular endothelial growth factor (VEGF) and microRNA-126, highlighting its regulatory role for modulation of diabetic heart disease [18]. Exercise training also ameliorates inflammation and oxidative stress in animal models with diabetic heart disease by enhancing the expression of superoxide dismutase 2 (SOD2) and endothelial nitric oxide synthase (eNOS), along with activation Nrf2-dependent antioxidant signaling pathway [19]. Moreover, physical training improves mitochondrial function and restores calcium handling in diabetic myocardium [20]. These findings are backed up by clinical evidence, as a review of type II diabetes patients has shown that exercise training improves systolic and diastolic function of the left ventricle [21]. Preclinical findings have demonstrated that exercise training triggers mitochondrial function in diabetic hearts, a concept strengthened by clinical evidence in men with obesity, where 12 weeks of circuit and high-intensity interval training (HIIT) increased the expression of adipokine Nrg4, leading to improved cardiometabolic risk profiles [22]. Despite clear benefits, diabetic patients often fail to engage in exercise due to limited awareness, physical and social constraints, inadequate infrastructure, low motivation, along with time limitations and minimal physician guidance [23].

Exercise training is an effective method in mitigating diabetic heart disease through improved glycemic control, mitochondrial function, calcium handling, and upregulation of growth factors. However, its effects on oxidative phosphorylation-related genes remained unclear. We hypothesized that exercise training is a potent modulator of mitochondrial oxidative phosphorylation-related genes, in turn enhancing energy metabolism of the diabetic heart. To clarify this hypothesis, we conducted this study aimed to: (1) identify major molecular pathways modulated by exercise training in the diabetic heart, apoptosis, diabetic cardiomyopathy, and oxidative phosphorylation, and (2) determine the specific transcriptional changes in genes of mitochondrial oxidative phosphorylation using RNA extraction and microarray analysis in diabetic mice subjected to structured exercise training.

## Methodology

### Animals

Twenty-four male C57BL/6J Narl mice of 8 weeks age were provided standard housing under controlled conditions (25 °C, 12-hour light/dark cycle), ad libitum access to standard chow and tap water. All animals were maintained in the approved animal facility of China Medical University, Taichung, Taiwan.

### Diabetes induction

Streptozotocin (STZ) (100 mg/kg body weight), freshly dissolved in 0.1 M sodium citrate buffer (pH 4.5), was used to induce diabetes based on a previously established protocol [24]. This dose is enough to cause diabetes by destruction of β-cells and maintenance of hyperglycemia (fasting blood glucose > 11.1 mmol/L or 200 mg/dL), along with reducing systemic toxicity and mortality. Fasting blood glucose was assessed 48 h post-injection from the blood of the tail vein using Roche Accu-Chek Soft test strips.

### Experimental groups

**Fig. 1:**
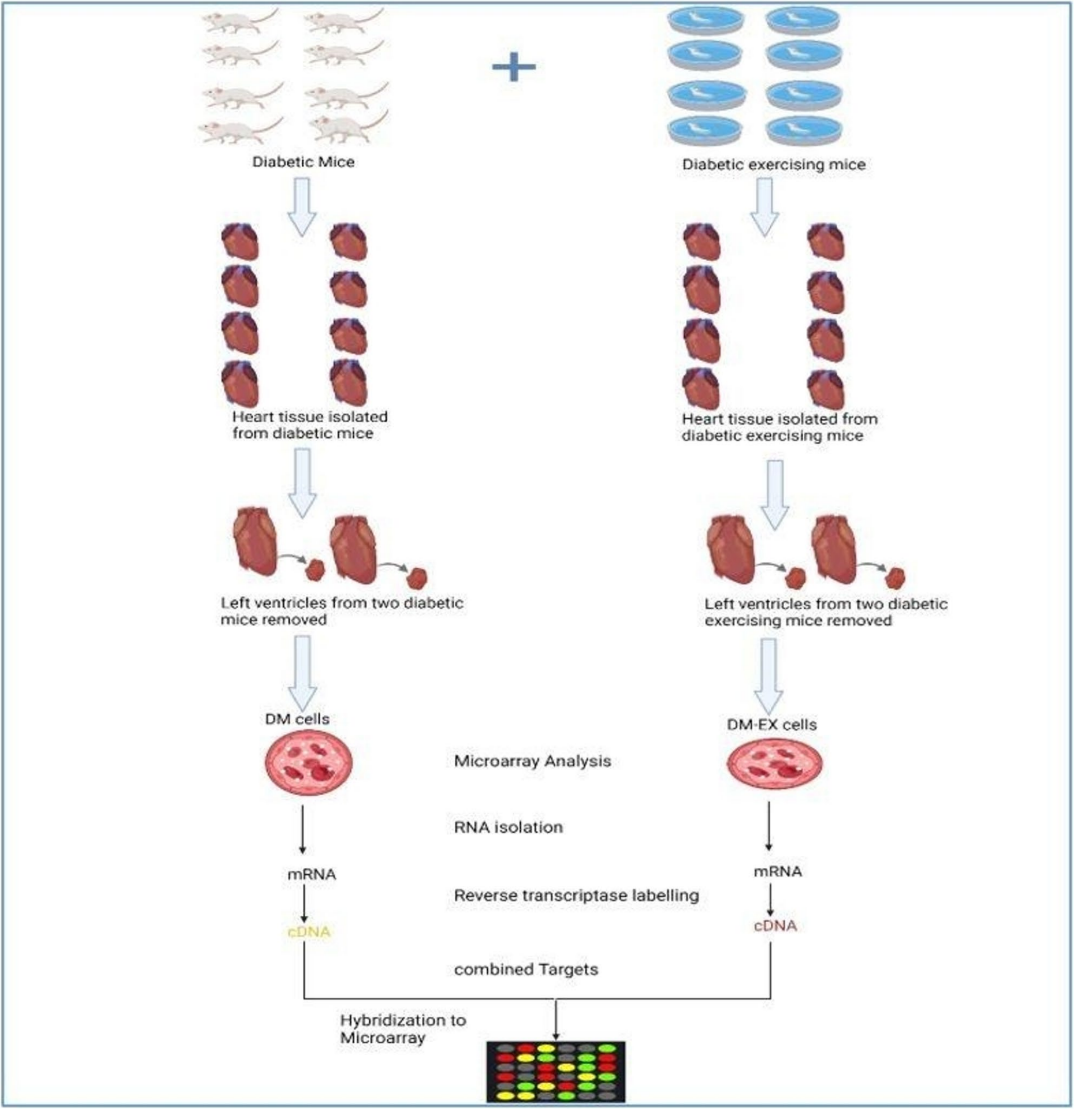
Experimental workflow for cardiac tissue RNA-seq and microarray analysis

Twenty-four male mice were randomly divided in to three groups (*n* = 8 per group): STZ-induced diabetic with swimming exercise (DM-EX); diabetic (DM); a non-diabetic control group, The swimming protocol was adapted from previous studies [24, 25] and selected as a well-validated, non-weight-bearing endurance exercise that elicits consistent cardiovascular and metabolic adaptations while minimizing stress compared with treadmill running [26]. DM-EX mice performed progressive endurance-type swimming in a 60 × 90 × 50 cm water tub sustained at 35 ± 1 °C. Exercise duration was 15 min/day, 5 days/week for the initial two weeks, increased to 20 min/day in the third week, and maintained at 30 min/day from weeks 4–12, representing low-to-moderate intensity in the first two weeks followed by moderate-intensity aerobic training from 3rd through twelve weeks. After each session, mice were gently dried to prevent hypothermia. After 48 h from the final exercise session, mice were anesthetized using CO₂ inhalation according to the IACUC guidelines, and no injectable anesthetics were used. After confirming anesthesia, the mice were then euthanized by decapitation to avoid acute exercise effects. Hearts and soleus muscles were harvested, and two left ventricles per DM and DM-EX group were randomly selected for RNA extraction and microarray analysis (Fig. 1).

### Citrate synthase activity

Soleus muscles (~ 0.1 g) were excised, frozen, and kept at -80 °C until analysis. At assay time, frozen tissues were homogenized on ice with a mechanical homogenizer (Model 398, Biospec Products, Mexico) in an ice-cold homogenization buffer. The homogenates were centrifuged at 13,000 rpm for 15 min at 4 °C, followed by collection of supernatants for enzymatic analysis. Citrate synthase activity was measured spectrophotometrically using the 5,5′-dithiobis (2-nitrobenzoic acid) (DTNB, Ellman’s reagent), coupled method, as reported before [27]. Briefly, 50 µL of tissue supernatant was mixed with acetyl-CoA (final concentration 10 mM; 30 µL) and distilled water (770 µl) to reach the reaction volume, then transferred to a cuvette containing DTNB (1 mM, 100 µL) in assay buffer. Reaction was stimulated by adding 50 µL of 10 mM oxaloacetate, and the increase in absorbance at 412 nm (formation of thionitrobenzoate, TNB) was recorded for 5–10 min. The rate of absorbance change in linear portion of the graph was used to calculate citrate synthase activity. Each sample was assayed in duplicate, and enzyme activity was normalized to total protein content. All procedures were conducted on ice or at 4 °C unless otherwise stated.

### RNA extraction

Cells were harvested by scraping and pelleted via centrifugation. Total RNA was acquired using the RNeasy Mini Kit (Qiagen, Cat. No. 74104) according to the manufacturer’s protocol. RNA purity and concentration were measured by absorbance at 260/280 nm using a NanoDrop ND-1000 spectrophotometer (Labtech International, UK). Microarray analysis was performed using 300 ng of total RNA from each sample, amplified and labelled by the GeneChip™ WT Sense Target Labeling and Control Reagents kit (Affymetrix, 900652).

### Microarray analysis

According to the manufacturer’s recommendations, the Affymetrix Gene Chip Human Gene 1.0 ST array was used to perform hybridization. Array’s hybridization was carried out for a total of 17 h, at 60 rpm and 45 °C hybridization. Following hybridization, arrays were cleaned (Affymetrix Fluidics Station 450) for staining with streptavidin-phycoerythrin (Gene Chip® Hybridization, Wash, and Stain Kit, 900720), and scanned using Affymetrix Gene Chip® Scanner 3000. 

### Statistical analysis

The microarray data were analyzed using the Limma package of the R language [28]. To maintain consistency across the datasets, background correction and quantile normalization were applied. A linear model along with empirical Bayes moderation was used for differential gene expression between groups, as it stabilizes variance estimates and improves statistical reliability, especially for small sample sizes. Benjamini-Hochberg false discovery rate (FDR) was applied for multiple testing correction to control type I errors inherent in high-throughput analyses. Genes meeting both the biological relevance and statistical significance (*p* < 0.05) were prioritized for downstream functional and pathway enrichment analyses.

### Bioinformatics and functional analysis

Gene expression data were further analyzed using R (R Core Team, 2020; RStudio, PBC, Boston, MA). To visualize the DEGs and identify similarity patterns in DM and DM-EX groups, heatmaps with hierarchical clustering were generated. To evaluate overall variance and reduce dimensionality, Principal Component Analysis (PCA) was conducted. To obtain biological pathways related to the DEGs, enrichment analysis was carried out, followed by KEGG pathway mapping to illustrate the distribution of these genes within the relevant molecular pathways [29].

## Results

### Effects of exercise training on myocardial properties of the mice

Body weight (BW), whole heart weight (WHW), and left ventricular weight (LVW) did not differ significantly between DM and DM-EX groups, whereas both groups exhibited lower body weight compared to the control group. Fasting blood glucose was markedly elevated in DM and DM-EX mice compared with controls (Table 1). Citrate synthase, a key marker of exercise effect, was measured in the soleus to assess the training efficacy. Exercise training significantly enhanced citrate synthase activity in DM-EX mice in relation to DM and control groups (Table 1), indicating enhanced mitochondrial oxidative capacity in response to exercise.

**Table 1:** Characteristics of diabetic mice with exercise training

	Control	DM	DM-EX
Number of mice	8	8	8
Body weight (BW), g	25.96 ± 2.59	21.37 ± 4.00 ^*^	22.20 ± 4.2 ^*NS^
Whole heart weight (WHW), g	0.139 ± 0.018	0.138 ± 0.020	0.129 ± 0.036 ^NS^
Left ventricular weight (LVW), g	0.101 ± 0.015	0.088 ± 0.015	0.089 ± 0.025 ^NS^
Glucose, mg/dL	166.6 ± 55.1	506.0 ± 55.0 ^**^	484.1 ± 91.1^**^^NS^
Citrate synthase activity, µmol/min/g/wt	1.82 ± 0.06	1.83 ± 0.07	2.32 ± 0.08^*#^

Values are presented as mean ± SD

Soleus citrate synthase activity indicate effects of exercise training

^*^p < 0.05, significant different from control, ^**^P < 0.01, significantly different from the control group

^NS^ non-significant different from DM

^#^*p* < 0.05 significant different from DM

### Gene expression analysis

**Fig. 2:**
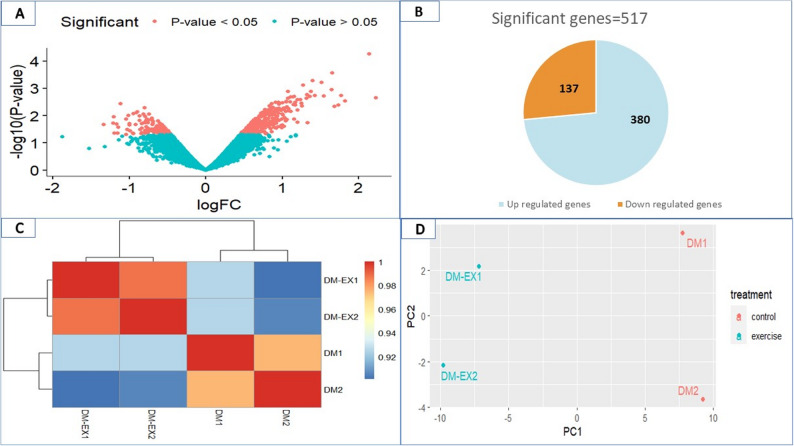
Comparative gene expression analysis of DM-EX and DM mice. **A** Volcano plot showing significant (orange, *p *< 0.05) and non-significant (green, *p *> 0.05) genes. **B** Number of significant genes: 517 total, with 380 upregulated and 137 downregulated in DM-EX versus DM. **C** Heatmap with hierarchical clustering, demonstrating the difference between DM-EX and DM groups. **D** Principal component analysis (PCA) exhibiting separated clustering of DM-EX and DM groups: PC1 (principal component 1); PC2 (principal component 2)

Volcano plot analysis illustrated significant and non-significant genes between DM-EX and DM mice (Fig. 2A), with significant genes (*p* < 0.05) highlighted in orange and non-significant genes (*p* > 0.05) in blue. 517 genes were significantly differentially expressed in DM-EX versus DM, including 380 upregulated and 137 downregulated (Fig. 2B). Quality control using heatmap visualization and principal component analysis (PCA) confirmed distinct transcriptomic profiles between groups. The heatmap showed clear clustering of DM-EX and DM samples into separate dendrograms (Fig. 2C), and PCA revealed consistent separation of the two groups, reflecting exercise-induced changes in gene expression (Fig. 2D).

### Functional enrichment analysis

**Fig. 3:**
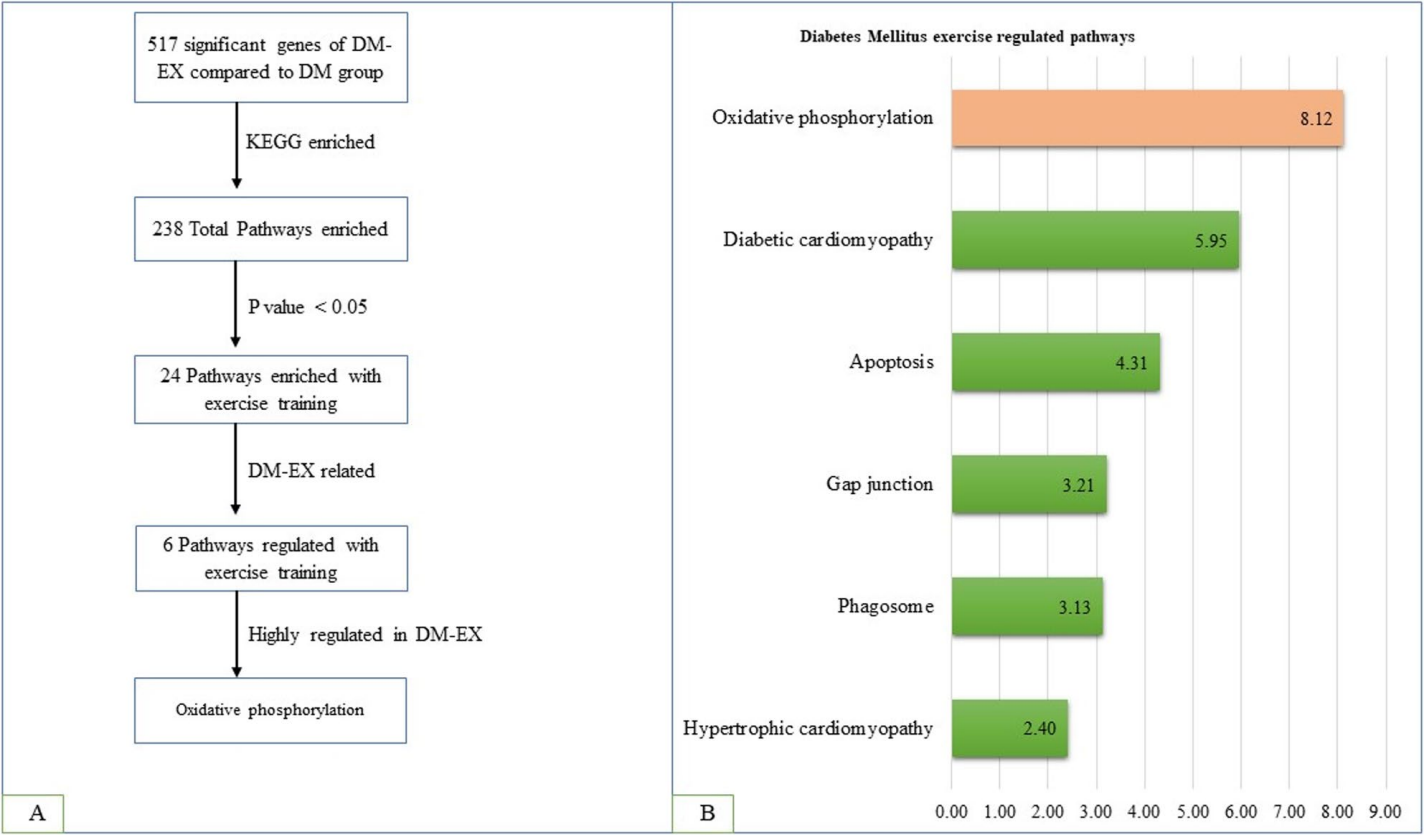
Enrichment analysis of differentially expressed genes in DM-EX versus DM mice. **A** KEGG pathway enrichment of significant genes as analyzed using Metascape. **B** Exercise-regulated pathways in the diabetic heart

DEGs identified in DM-EX versus DM mice were further analyzed using Metascape (Fig. 3A). KEGG pathway enrichment identified 238 pathways, of which 24 were significantly regulated by exercise training (*p* < 0.05). Pathways were manually curated to determine exercise-responsive pathways in diabetic heart: oxidative phosphorylation, diabetic cardiomyopathy, apoptosis, gap junction, phagosome, and hypertrophic cardiomyopathy (Fig. 3B). The top three most strongly regulated pathways were oxidative phosphorylation, diabetic cardiomyopathy, and apoptosis, with oxidative phosphorylation exhibiting the most significant regulation. 

### Exercise training enhanced mitochondrial oxidative phosphorylation gene expression

**Fig. 4:**
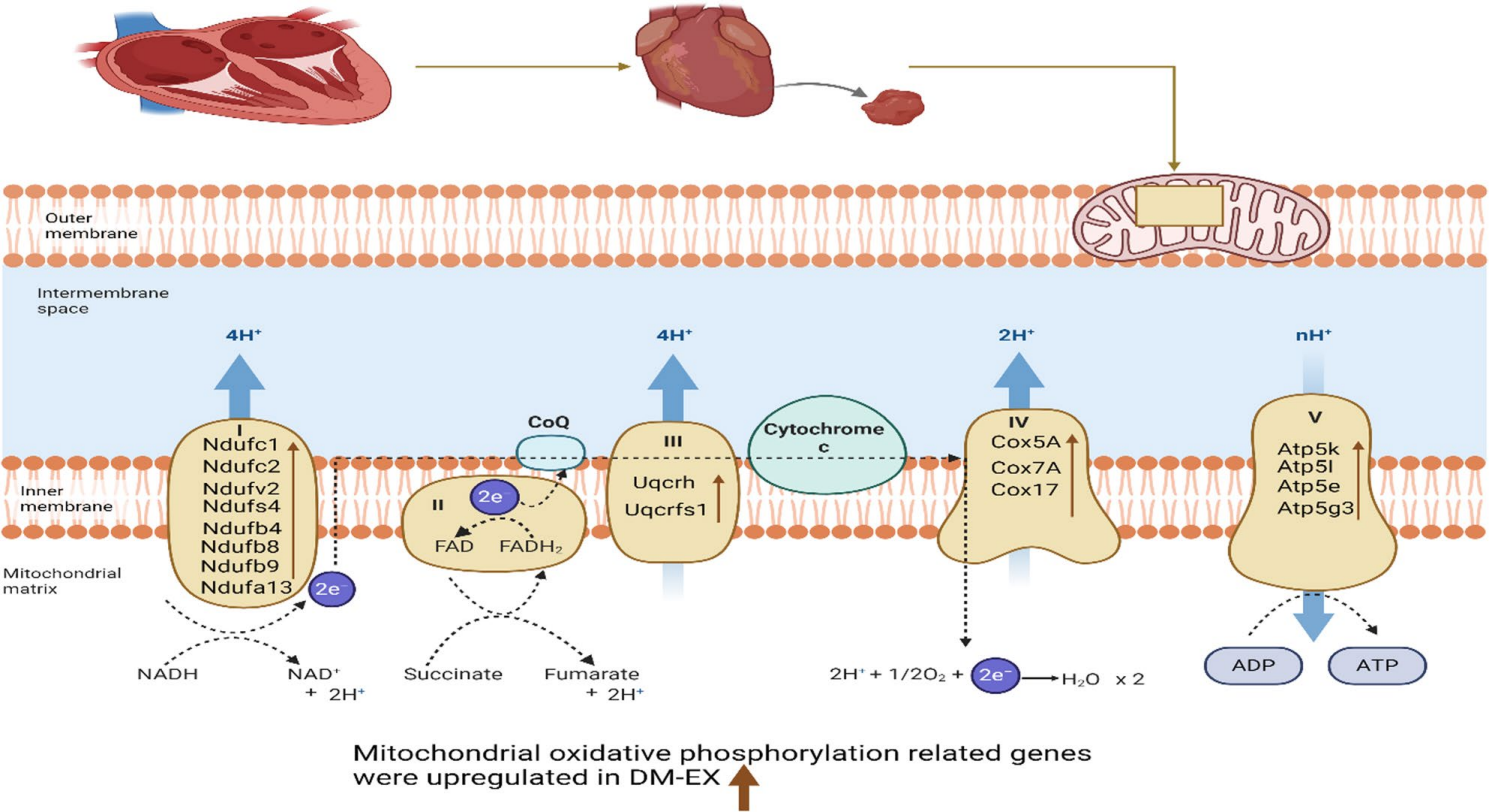
Exercise training enhances the expression of oxidative phosphorylation genes in the diabetic heart

Exercise training significantly upregulated genes associated with mitochondrial oxidative phosphorylation across ETC complexes. In Complex I, the expression of Ndufb8, Ndufb4, Ndufc2, Ndufv2, Ndufs4, Ndufa13, Ndufb9, and Ndufc1 was elevated in response to exercise. Key genes in Complex III (Uqcrh, Uqcrfs1) and Complex IV (Cox17, Cox5A, Cox7A) were similarly upregulated. Moreover, genes of Complex V, including Atp5l, Atp5k, Atp5e, and Atp5g3, showed enhanced expression following exercise training (Fig. 4). These results indicate that exercise enhances the transcriptional regulation of mitochondrial components critical for energy production in diabetic hearts.

## Discussion

This study aimed to identify the key molecular pathways regulated by exercise training in the diabetic heart. Exercise training influenced multiple pathways in the diabetic heart, such as those related to apoptosis, diabetic cardiomyopathy, phagosome function, hypertrophic cardiomyopathy, gap junction, and oxidative phosphorylation, identified as the most significantly regulated pathway. Notably, exercise training upregulated key oxidative phosphorylation related genes across various mitochondrial complexes I and III-V, including Ndufc1, Ndufc2, Ndufv2, Ndufs4 Ndufb4, Ndufb8, Ndufb9, and Ndufa13 (Complex I); Uqcrh and Uqcrfs1 (Complex III); Cox5A, Cox7A and Cox17 (Complex IV); and Atp5k, Atp5l, Atp5e, and Atp5g3 (Complex V), demonstrating enhanced mitochondrial metabolism and oxidative phosphorylation in diabetic heart (Fig. 4).

Our findings demonstrated that exercise increased the expression of mitochondrial oxidative phosphorylation genes and maintained myocardial energy homeostasis, thereby improving myocardial function in diabetic heart. Mechanistically, exercise training enhanced the stimulation of PGC-1α and Akt, followed by enhanced expression of downstream transcriptional factors TFAM, NRF1, and mtDNA, thereby enhancing mitochondrial oxidative phosphorylation and metabolic efficiency in diabetic cardiomyopathy [30]. Exercise training also stimulates AMPK/PGC-1α signaling pathway, which is disturbed in diabetic hearts, in turn thereby improving mitochondrial biogenesis and energy metabolism, along with restoring myocardial energetic homeostasis [31]. Moreover, exercise training enhances the expression of the SIRT1-AMPK-PGC-1α axis, while suppressing NFκB signaling, further improving mitochondrial oxidative phosphorylation and biogenesis, in diabetic heart [32]. By stimulating AMPK and SIRT1, exercise enhances PGC-1α deacetylation and transcriptional activity, upregulating nuclear-encoded mitochondrial genes such as those of complexes I, which likely underlie the observed improvements in oxidative phosphorylation gene expression. Through activation of AMPK and SIRT1, exercise enhances PGC-1α deacetylation and transcriptional activity, upregulating nuclear-encoded mitochondrial genes such as those of complexes I, which likely underlie the observed improvements in oxidative phosphorylation gene expression.

Animal models of type I diabetes exhibit markedly reduced mitochondrial oxidative phosphorylation complex activity, accompanied by a coordinated downregulation of genes encoding these complexes [33]. Streptozotocin-induced diabetic rat hearts exhibit enhanced mitochondrial lysine acetylation due to excessive oxidation of fatty acid oxidation and a defect of Complex I, which diminishes the function of ETC and compromises mitochondrial oxidative phosphorylation [34]. In diabetic hearts, an imbalance of complex I subunits, notably NDUFB8, arises from SIRT1-mediated deacetylation [35], while knockdown of NDUFC2 and NDUFS4 significantly downregulates metabolic and oxidative phosphorylation pathways, exacerbating diabetic cardiomyopathy [36]. Hyperglycemia also decreases NDUFA13 expression and increases reactive oxygen species production in H9C2 cardiomyocytes [37]. Additional studies show downregulation or mutation of NDUFB4, NDUFV2, and NDUFB9 in models of cardiovascular disease and diabetic cardiomyopathy, highlighting the vulnerability of complex I subunits to metabolic stress [38–41]. Exercise training consistently counteracts these impairments; Our study demonstrated upregulation of NDUFB8, NDUFB4, NDUFC2, NDUFV2, NDUFS4, NDUFA13, NDUFB9, and NDUFC1 in diabetic hearts. These findings align with evidence that physical activity regulates NDUFB8 and NDUFB9 in the aging hippocampus [42], hypomethylates NDUFC2 in skeletal muscle and downregulates NDUFC1 in vastus lateralis [43, 44], and increases NDUFB4 and NDUFV2 expression in mouse hearts following endurance or resistance training [45, 46].

Hyperglycemia impairs Complex III and IV activity in neonatal rat cardiomyocytes through O-GlcNAcylation in core 1 and 2 subunits of Complex III and subunit I of Complex IV [47]. This downregulation of Complex III genes, including UQCRH and UQCRFS1, and Complex IV genes, such as COX5A, COX7A, and COX17, disrupts electron transport, reduces ATP production, increases ROS, and contributes to impaired cardiac contractility, structural remodeling, and bioenergetic decline, thereby accelerating diabetic cardiomyopathy [48–52]. Our data suggested that exercise training upregulates gene expression in both Complex III (UQCRH, UQCRFS1) and Complex IV (COX5A, COX7A, COX17) in the diabetic heart. This aligns well with the previous reports demonstrating that resistance training counteracts aging by upregulating genes such as UQCRH and UQCRFS1, with effects that are detectable at both phenotypic and transcriptomic levels [53]. Similarly, exercise training enhances VO₂ max and type I fiber proportion, accompanied by increased COX5A expression in aging populations, while exerting no significant effect on COX17 levels in plasma exosomes of diabetic mice [53, 54].

Complex V, the final component of the electron transport chain, generates ATP from ADP in the mitochondrial matrix using the proton electrochemical gradient [55]. In diabetic mice, mitochondrial uncoupling and increased proton leak reduce ATP production [12], accompanied by downregulation of oxidative phosphorylation genes, including ATP5K, ATP5L, and ATP5E [56, 57]. Our study demonstrates that exercise training upregulates the expression of complex V genes ATP5K, ATP5L, ATP5E, and ATP5G3 in the diabetic heart. Previous studies using proteomic analyses have shown that high-intensity interval training upregulates ATP5L but not ATP5E in human skeletal muscle [58], highlighting both consistency and contrast with our findings in the diabetic heart. Voluntary wheel running increases ATP5K in mice, promoting physiological hypertrophy, while high-intensity interval training upregulates ATP5G3 in human skeletal muscle [59, 60]. Collectively, exercise training markedly increases Complex V gene expression and, together with upregulation of Complexes I, III, and IV, enhances oxidative phosphorylation and mitochondrial morphology, potentially mitigating diabetic cardiac dysfunction.

Translating these findings to humans, 12 weeks of HIIT or circuit resistance training in obese men increased adipokine Nrg4 and improved cardiometabolic risk factors, whereas moderate-intensity training was less effective [22]. In obese males, Interval resistance training at moderate and high intensities also enhanced myokines (decorin, follistatin, myostatin, TGF-β1) and cardiovascular risk factors compared to low intensity [61]. Similarly, 12 weeks of resistance and aerobic exercise improved adipokines (lipocalin-2 increased, omentin-1 decreased), insulin resistance, and body composition in diabetes [62]. Exercise training reduced the expression of TNF-α, IL-6, and elevated adiponectin in type II diabetic patients [63], while HIIT downregulated p53, enhanced mtDNA and COX, improved oxidative stress markers and glycemic control [64]. These reports highlight the role of exercise intensity in driving molecular adaptations in diabetes. However, translation to humans should be cautious, as the interspecies difference in mitochondrial turnover, cardiac physiology, and gene expression dynamics may impact the magnitude and timing of these adaptations [65]. Notably, direct assessment of cardiac mitochondrial oxidative phosphorylation-related gene expression in humans is lacking, highlighting the need for targeted clinical trials.

### Limitations

This study has several limitations. Firstly, despite standard housing, diet, and exercise training, some potential confounding factors, such as the composition of the diet, severity and duration of diabetes, type and intensity of exercise, as well as handling or water-induced stress, cannot be completely excluded. Secondly, we acknowledge that the sample size (*n* = 2 per group) is small and has constraints for statistical power. Still, the use of Limma moderated statistics and pathway-level analyses offered a sound framework for identifying biologically meaningful patterns. Thirdly, direct comparisons of genes in relation to exercise in the diabetic heart were challenging due to the limited literature available regarding exercise-induced regulation of mitochondrial and oxidative phosphorylation genes in the diabetic heart.

## Conclusion

Exercise training resulted in 517 DEGs; 380 upregulated, 137 downregulated, influencing pathways such as diabetic cardiomyopathy, apoptosis, and oxidative phosphorylation. Exercise training upregulated 17 key oxidative phosphorylation-related genes across complexes I, III, IV, and V. These results signify the role of exercise training in mitochondrial bioenergetics and cardiac adaptations. Future studies should focus on exploring different exercise types and durations to maximize mitochondrial and myocardial metabolic parameters, providing a translational framework to develop targeted exercise therapy for the prevention of diabetic heart disease in humans.

## Data Availability

The raw data supporting the findings of this study will be provided by the corresponding author ( [shinda@mail.cmu.edu.tw](mailto: shinda@mail.cmu.edu.tw) ) upon reasonable request.
